# Road bike accidents involving cervical fractures presenting as cardiac arrest: a report of two cases

**DOI:** 10.1186/s13049-019-0641-3

**Published:** 2019-07-08

**Authors:** Anne Marie Kabell Nissen, Louise Gramstrup Nielsen, Søren Mikkelsen, Anne Craveiro Brøchner

**Affiliations:** 1 Department of Anaesthesiology and Intensive Care Medicine, Odense University Hospital, 5000 Odense, DK Denmark; 2 The Prehospital Research Unit, Region of Southern Denmark, Odense University Hospital, Odense, Denmark; 3 Department of Regional Health Research, University of Southern Denmark, Odense, Denmark; 4 Kolding Hospital, a part of Hospital Lillebaelt, Kolding, Denmark

**Keywords:** Cervical spine fracture, Cardiac arrest

## Abstract

We present two cases in which elderly male recreational cyclists suffered from cervical fractures and coinciding injuries of the spinal cord that subsequently led to cardiac arrest.

Based on reports from eye witnesses and due to the low impact nature of the crashes, the two patients were initially considered as having cardiac arrest before falling of their bikes.

The spinal cord injuries triggering cardiac arrest were acknowledged with delay, as the primary eliciting cause was considered cardiac disease in conjunction with all-out exercise. We suggest that increased focus should be made on possible cervical injuries even following low energy crashes in road cycling.

## Background

Middle-aged or elderly men performing recreational cycling are over-represented in the group of patients presenting with exercise-related severe cardiovascular events [1]. Thus, in general, it is reasonable to assume that in this population of cyclists, the eliciting cause is exercise-related, when they are found with cardiac arrest on the road next to the bicycle.

This is, however, not always the case.

Falls and other accidents were previously primarily seen in off road cycling (mountain biking), mostly resulting in minor injuries [2, 3]. However, in recent years, there has been an increase in injuries, as off road cycling appears to be a high-risk sport with regard to severe spine injuries [4]. It has been reported that cycling-related spine injuries are more frequent in off road cycling compared to road cycling [5]. Although cervical cord injury (CCI) in cycling accidents not involving vehicles is rare [6], cycling has been shown to be the primary cause of traumatic cervical fractures in a sports-related sub-population [7].

CCI is reported to occur in 10 to 50% of traumatic cervical fractures [8]. Following CCI, bradycardia is the most common cardiac arrhythmia. Cardiac arrest following CCI has both been described immediately after CCI and in the days following CCI [9].

We present two cases of middle-aged or elderly men with suspected exercised-induced cardiac arrest following seemingly innocent falls in recreational road cycling. Both men were later diagnosed with cervical fractures, CCI being identified as the eliciting cause of cardiac arrest.

## Case presentation

A 68-year-old man was admitted to hospital following cardiac arrest during indoor track cycling. Bystanders described the events leading up to the cardiac arrest as the cyclist gradually losing speed, eventually falling sideways off the bike. This suggested to the attending prehospital anaesthesiologist that the cyclist got ill before he actually fell of the bike. No obvious signs of trauma were noted and the helmet remained intact. Bystanders quickly acknowledged that the patient had cardiac arrest and initiated resuscitation efforts. An automatic electronic defibrillator was attached just as the prehospital anaesthesiologist and the ambulance arrived. The initial rhythm analysis revealed pulseless electric activity. Following three to 4 minutes of treatment for cardiac arrest, return of spontaneous circulation was achieved. However, spontaneous respiration did not return.

The patient was intubated at the scene and escorted to the regional university hospital as exercise-related cardiac arrest was suspected. At the hospital, a fellow bicyclist eventually revealed that the patient in fact was hit by another bicycle rider immediately before the crash. Therefore, the initial hypothesis that the cyclist became ill before the fall was discarded and trauma was suspected. The patient had a computerised tomography (CT) scanning performed, which revealed an isolated fracture of dens axis type 2 and contusion of the medulla oblongata at the affected level.

Cardiac genesis was excluded based on results from echocardiography, electrocardiography, and blood samples including Troponin I.

The following day the patient showed signs of spinal shock and autonomic dysfunction, including bradycardia and asystole, prompting placement of a pacemaker. Repeated electroencephalograms revealed refractory myoclonic status epilepticus. As the patient did not regain consciousness, treatment was withheld 6 days after the accident, and the patient deceased shortly afterwards.

The second case was a 73-year-old man who was admitted to a cardiology department after being resuscitated from cardiac arrest during a road bike race. Bystanders described the man wobbling on the bike, eventually falling in a ditch. Cardiac arrest was quickly acknowledged and resuscitative efforts initiated. Approximately 5 minutes of CPR was given.

At the arrival of the pre-hospital emergency caretakers, the patient had regained spontaneous circulation and respiration. No medicine or shock had been given.

Pre-hospital electrocardiography revealed no signs of cardiac ischemia. The patient, now awake, had no complaints, be it chest, back or neck pain. The patient was referred to the cardiology department as the primary diagnosis assigned to the patient was exercise-related cardiac arrest.

Upon arrival at the department of cardiology, echocardiography revealed a preserved ejection fraction and no regional hypokinesia of the heart.

After cardiac examinations, the patient now began to complain of neck pain. A CT scanning was performed revealing a dislocated fracture of dens axis.

The patient was immediately inline-stabilised and transferred to the trauma unit at the regional university hospital. He was admitted at the neurosurgical department, presenting no signs of autonomic dysfunction. Four days later he underwent a successful operation to stabilize the cervical column. The patient eventually achieved full recovery.

During hospital admission the patient was consulted by a cardiologist ruling out primary cardiac event. The patient stated that there were no prodromal events prior to the accident. He simply had steered too close to the edge of the roadside ditch and subsequently crashed.

Although the cause of cardiac arrest could not be established with complete certainty, no other plausible cause than acute CCI could be found.

## Discussion and conclusion

Exercise-related severe cardiac events such as coronary occlusion and cardiac arrest are feared complications in middle-aged or elderly patients performing all-out exercise. Coronary heart disease is responsible for most of these events, and cycling is the most common sport associated with these cardiovascular events [1].

In the cases presented, the common denominator was the assumption that cardiac arrest preceded the crashes, and both patients were initially treated as patients with primary cardiac arrest. In both cases the cervical trauma and CCI was acknowledged with delay.

CCI’s effect on the cardiovascular system is due to the disruption between the autonomic centres in the brain and the sympathetic neurons in the spinal cord. This results in a complete lack of sympathetic tonus causing vasodilatation and bradycardia; concurrently, an unopposed vagal tone activates the parasympathetic nervous system. This combined autonomic dysfunction can lead to severe bradycardia and asystole.

It has previously been reported, that regardless of performing road cycling or off road cycling, severely injured cyclists display similar patterns of injury and comparable outcomes following accidents. The exception is spinal injuries [5], and concern has been raised in regards to spinal injuries following crashes in off road cycling [10].

It is a common conception that off road cycling is riskier than road cycling as crashes in general may be considered high-energy crashes, potentially involving cliffs, hillsides and trees. As such, appropriate diagnostic measures regarding CCI are often made when caring for an acutely injured mountain bike cyclist following a crash. This conception, however, may be wrong. It has been reported that road-biking is more often associated with head-trauma, whereas off road cycling causes a greater number of less severe injuries [11].

Crashes of seemingly low intensity may also be harmful. The incidence of traumatic cervical fracture in low-energy trauma has been investigated in a study from Norway. The study concluded that 57% of traumatic cervical fractures were caused by falls from less than 1 m or less than 5 stair-steps [12]. Hence, it is probably not correct to assume that the degree of energy in the trauma is always proportional to the incidence of traumatic cervical fractures. This may have implications for road cyclists or indoor track cyclists.

An increase in spinal injuries has been linked to a growing popularity of cycling, and in particular competitive cycling [13]. Both the emergency medical systems and the emergency departments should thus increase focus on spinal cord injuries in all forms of recreational cycling.

The two cases presented here are reported from two different prehospital units and two different hospitals in the Region of Southern Denmark. Both cases demonstrate that considerations pertaining to traumatic injury are not necessarily the first considerations made when cardiac arrest is encountered in conjunction with what may be perceived as low-energy trauma.

## Conclusion

With the presentation of these two cases, we highlight that cardiac arrest in recreational road cyclist may be caused by CCI following crashes. CCI should thus be suspected following crashes regardless of the immediate reports on events leading up to the incident given by bystanders. We suggest that appropriate measures regarding spinal stabilisation should be made in patients with cardiac arrest even following seemingly minor accidents with potential for spinal cord injury.

## Data Availability

Data sharing not applicable to this article as no datasets were generated or analysed during the current study.

## Abbreviations

CCI: Cervical cord injury
CPR: Cardiopulmonary resuscitation
CT: Computerised tomography
